# Uncertainty-aware estimation, planning, and control for tracking multiple drifting patches in flow fields

**DOI:** 10.3389/frobt.2026.1842384

**Published:** 2026-06-26

**Authors:** Daniel O. Akanji, Krishnanand N. Kaipa, Cong Wei

**Affiliations:** Department of Mechanical and Aerospace Engineering, Old Dominion University, Norfolk, VA, United States

**Keywords:** autonomous marine vehicles, boundary fusion, flow-field estimation, HF-radar replay, LQR, patch tracking, persistent monitoring, receding-horizon scheduling

## Abstract

In this study, we present a replay-based framework for uncertainty-aware persistent tracking of multiple advected surface patches using an autonomous marine vehicle operating in spatiotemporal-varying currents. The method combines three components: local flow estimation, covariance-aware patch-boundary propagation with intermittent boundary fusion, and mission-level scheduling over multiple patches. Each patch is represented by a polygonal boundary, whose vertices are propagated through the estimated flow field while carrying per-vertex covariance, thereby quantifying uncertainty growth during advection. A flow-aware gain-scheduled linear quadratic regulator (LQR) was designed to shape the desired surge speed to take advantage of favorable currents. When the vehicle services a patch, boundary detections are fused to reduce the active patch uncertainty, and optional local map-covariance refinement is used to reduce subsequent uncertainty regrowth in the surrounding flow field. A boundedness analysis shows that if each patch is revisited within a prescribed maximum interval, then the corresponding patch uncertainty remains uniformly bounded; a companion feasibility condition relates the allowable revisit interval to vehicle speed, service time, and tour length over the patch set. To validate the result, a data replay simulation using HF-radar currents from the San Francisco Bay region was used to demonstrate the expected bounded sawtooth uncertainty behavior under feasible revisit conditions. In addition, our proposed duration-weighted predictive scheduler outperforms nearest-patch and round-robin baselines and, in spatially separated patch configurations, achieves lower mean patch uncertainty and lower control-effort proxy than a highest-J baseline. These results indicate that combining uncertainty-aware propagation with cost-aware scheduling is a viable strategy for persistent monitoring of evolving marine surface phenomena.

## 1 Introduction

In this study, we present a framework for an autonomous vehicle to track multiple drifting surface patches by propagating the patches. The pollution in rivers and coastal waters, including pollution plumes, algal blooms, marine debris, garbage patches, and oil spills, poses significant threats to the environment and can be toxic to humans (Ma et al., 2025; Anderson et al., 2002; Lebreton et al., 2018; Peterson et al., 2003). Spatio-temporally varying flow fields (currents and eddies) are a dominant factor in the dispersion, deformation, and movement of these dynamic phenomena, making static survey patterns unable to capture their dynamics (Okubo, 1971; McWilliams, 2016). As a result, preplanned transects can waste time and energy surveying empty water and may fail to repeatedly revisit the most informative parts of a moving feature. Autonomous marine robots are increasingly being used for environmental monitoring, ocean sampling, and ecological surveys (Peck et al., 2024; Hwang et al., 2019). These systems have advanced scientific capabilities in understanding the coastal and ocean environments. Although these vehicles can operate in this harsh and unpredictable environment, vehicle motion, sensor data interpretation, state estimation, trajectory tracking, and mission scheduling can be complicated by flow disturbances (Kinsey et al., 2006; Fiorelli et al., 2006). This necessitates the need for integrated estimation and control strategies that can leverage environmental information for robust navigation, mapping, and mission-level decisions (Knizhnik et al., 2022; Song and Mohseni, 2019).

Prior work has explored several aspects of flow-aware navigation and environmental tracking. Flow-informed planning strategies have demonstrated that predictions of water currents can be used to guide autonomous underwater vehicles toward the evolving features of interest. Notably, Smith et al. (2010) proposed trajectory planning methods that leverage predictions from a regional ocean model to track dynamic ocean processes. Although it shows the value of coupling environmental forecasts with vehicle motion planning, its dependence on external model infrastructure and forecast availability, which may not hold in riverine or communication-limited deployments, introduces associated challenges. Related approaches have focused on combining flow map estimation with state estimation to improve navigation performance. Song and Mohseni (2019) concurrently estimated robot pose and background flow field, showing how flow can aid SLAM. Flow-based control in gyre environments takes advantage of the spatial–temporal layout of gyres to aid persistent patrolling (Knizhnik et al., 2022).

In other environmental monitoring studies, researchers studied tracking and sampling of dynamic phenomena such as plumes, fronts, and diffusive fields. Petillo and Schmidt (2012) used multiple autonomous underwater vehicles (AUVs) to track underwater plumes. The type of plume, the spatial extent of the plume, and the AUV design were factors considered during mission planning. To handle numerous missions at the same time, adaptive autonomy to track the plume with the aid of a fleet of AUVs is developed (Petillo and Schmidt, 2012). Hwang et al. (2020) developed an AUV-based oil-plume mapping strategy that combines adaptive tracking and adaptive sampling using *in situ* acoustic measurements. Their method is especially relevant because it considers irregular plume geometries and adaptive vehicle responses to plume proximity and detection validity. Recent river-plume studies have also advanced real-time adaptive sampling and data-driven monitoring. This is evident in the study of Berild et al. (2024), demonstrating efficient real-time AUV adaptive sampling of a river plume front using onboard statistical surrogate modeling. Ge et al. (2025) proposed a long-horizon ${RRT}^{*}$ adaptive-sampling planner based on a cost-valley objective for sampling river-plume fronts, including real-time onboard implementation in a field trial. More recently, Fabbro et al. (2026) investigated multi-agent reinforcement learning for long-term river plume mapping using spatiotemporal Gaussian-process regression and ocean model simulations. These studies show the increasing importance of adaptive and data-driven autonomy for monitoring dynamic plume environments.

Although these studies provide important foundations, their primary objectives are often plume detection, scalar-field reconstruction, front/gradient tracking, or learned multi-agent sampling policies. Some of them treat the feature of interest as static between planning cycles. In contrast, the present study focuses on persistent tracking of multiple drifting patch boundaries using a single vehicle. Rather than treating the plume or patch only as a scalar field to be sampled, we maintain explicit polygonal boundary states, propagate per-vertex uncertainty under the estimated flow, fuse intermittent boundary observations, and schedule revisits based on predicted uncertainty growth and travel effort. Boundary-following methods, such as those proposed by Cruz and Matos (2014), are relevant when the goal is to remain near a salient front. Still, they do not explicitly maintain and schedule multiple uncertain advected patch boundaries.

In parallel, informative path planning (IPP) formalizes the problem of maximizing an information objective under cost budgets (energy/time) and has been applied to AUVs (Binney et al., 2010). Similarly, Ma et al. (2016) incorporated disturbance to explicitly account for ocean currents during planning and long-term monitoring. Yet, existing approaches largely treat estimation, tracking, and planning/scheduling as separate problems. In many cases, the planning objective is defined over a map or a scalar field directly with little emphasis on (i) maintaining and propagating multiple moving feature boundaries with uncertainty, (ii) fusing sporadic boundary observations into those boundaries, and (iii) planning which feature to service next using an energy-aware score that respects advection.

To address these gaps, we present an integrated framework for flow-aware patch tracking and multi-patch scheduling using a single autonomous vehicle. We represent environmental features as spatial patches that drift along with the ambient flow field. Surface patches in the ocean (e.g., oil slicks, algal blooms, or floating debris) are commonly modeled in a Lagrangian framework, in which boundary points (or particles) are advected by the near-surface velocity field, while unresolved processes introduce dispersion and modeling error in their boundary evolution (van Sebille et al., 2018). In practice, even if a mean current field is available, the patch perimeter undergoes stretching and filamentation due to spatial variability in the flow, and its predicted location becomes increasingly uncertain with time when unobserved. Motivated by these established physical and modeling practices, we represent each patch boundary by a fixed set of Lagrangian vertices. A state estimator, extended Kalman filter (EKF), is used to jointly estimate the vehicle state and a local flow map. The linearized local flow map is then used to propagate the associated uncertainty with additive process noise to capture unresolved variability. A flow-aware tracking controller exploits the estimated flow to guide the vehicle toward selected patch reference points while minimizing control effort. At a higher level, a multi-patch scheduler predicts the arrival uncertainty associated with each patch and the energy required to reach it, selecting targets that maximize expected benefit per unit cost.

The proposed framework naturally has a two-time-scale structure. At the fast time scale, the vehicle state, local flow estimate, patch-boundary propagation, and patch-reference tracking controller are updated at the estimation/control period Δt , while at the slower mission time scale, the scheduler is updated every $T_{\text{sch}}$ seconds to decide which patch should be serviced next based on predicted uncertainty growth and travel-energy cost. Recent work on mixed-dynamics and multi-time-scale robotic systems, including the dynamic confined space of velocities (DCSV) perspective, has emphasized that fast local dynamics and slower decision or constraint processes should not always be treated as homogeneous (Peña Fernández, 2022; Peña Fernández, 2023; Peña Fernández, 2026). Although the present study does not adopt DCSV as a formal control-allocation or configuration-selection framework, it is related in the sense that the proposed architecture also separates fast vehicle-level estimation, patch propagation, and tracking control from slower mission-level scheduling and intermittent service-event corrections. In this study, the revisit-time condition and scheduler score play the role of an operational constraint: they determine when the vehicle must return to each patch so that uncertainty growth remains bounded while respecting travel cost, service time, and vehicle mobility limits. Hence, our main contributions are as follows:

- Uncertainty-aware patch advection and flow coupling. We introduce a polygonal patch propagation model that advects boundary vertices under the ambient flow while propagating per-vertex covariance through local flow linearization, explicitly incorporating uncertainty from the estimated flow map.
- Lightweight boundary fusion for drift correction. We develop a patch-level boundary assimilation mechanism that aligns intermittent boundary detections with predicted patch geometry to correct advection drift, without requiring dense global mapping or mesh reconstruction. In addition, boundary displacements are used to locally refine the estimated flow map along the observed patch boundary.
- Uncertainty-aware multi-patch planning. We present a receding-horizon scheduling framework that selects which patch to service based on predicted arrival uncertainty relative to travel energy, enabling persistent and energy-efficient monitoring of multiple drifting processes.
- Flow-aware service-point tracking. We design an energy-aware gain-scheduled linear quadratic regulator (LQR) controller for tracking the selected patch reference under ambient flow. This links flow-assisted feed-forward control with the proposed multi-patch scheduling strategy.

The remainder of the paper is organized as follows. Section 2 formulates the problem in clear terms and the assumptions for which the derivation holds. Section 3 details the state and flow estimation algorithm, patch propagation and boundary fusion, and the patch-reference tracking controller, along with the multi-patch planning framework. In Section 4, we present and discuss the validation results for the framework, along with the performance metrics. We conclude the study in Section 5 detailing future improvements.

## 2 Problem formulation

We consider a single autonomous marine vehicle tasked with persistently monitoring multiple drifting surface patches in a horizontal flow field. A patch may represent a visible or measurable environmental feature such as a surface plume, algal bloom, oil slick, or floating debris region. The objective is persistent tracking and servicing of candidate patches after an external detection or survey stage has provided initial locations of the patches.

The main difficulty is that the patch states, vehicle state, and mission decisions evolve together but on different operational scales. Hence, each unattended patch continues to drift and deform under the flow field, causing its boundary uncertainty to grow. The vehicle has to service one patch at a time to reduce its uncertainty, which also delays its visits to the remaining patches. At the same time, the travel effort required to reach a patch depends on the vehicle location, the patch motion, and the surrounding flow. Therefore, the vehicle must repeatedly decide which patch should be serviced next so that uncertainty growth remains controlled while travel and control effort remain feasible. Thus, the problem is addressed through five coupled tasks:

1. estimating the vehicle state and local flow;
2. propagating each active patch boundary and covariance under the estimated flow;
3. fusing intermittent boundary observations at service events;
4. selecting which patch to service next using predicted uncertainty and travel effort; and
5. tracking the selected patch reference using a flow-aware controller.

### 2.1 Vehicle representation

Two reference frames are used. The inertial frame {n} has horizontal axes aligned with North and East, while the body-fixed frame {b} is attached to the vehicle center of gravity. The vehicle pose and body-frame velocity are defined as follows:

$$\eta ={\left[\begin{array}{ccc} x & y & \psi \end{array}\right]}^{⊤},\;\nu ={\left[\begin{array}{ccc} u & v & r \end{array}\right]}^{⊤},$$

where (x,y) is the horizontal inertial position, ψ is the yaw angle, and u , v , and r are surge velocity, sway velocity, and yaw rate, respectively.

The rotation from the body-fixed frame to the inertial frame is denoted by

$$R_{b}^{n}\left(\psi \right)=\left[\begin{array}{ccc} \cos\psi & -\sin\psi & 0\\ \sin\psi & \cos\psi & 0\\ 0 & 0 & 1 \end{array}\right],$$

and the inverse rotation is

$$R_{n}^{b}\left(\psi \right)={\left(R_{b}^{n}\left(\psi \right)\right)}^{⊤}.$$

The vehicle is modeled using standard planar 3-DOF surge–sway–yaw marine vehicle notation following Fossen (2011). The compact kinematic and dynamic models are presented as follows:

$$\dot{\eta }=R_{b}^{n}\left(\psi \right)\nu ,$$

$$M\dot{\nu }+C\left(\nu \right)\nu +D\left(\nu_{r}\right)\nu_{r}=\tau ,$$

respectively. Here, M is the inertia and added-mass matrix, C(ν) is the Coriolis–centripetal matrix, $D\left(\nu_{r}\right)$ is the hydrodynamic damping term, and τ is the generalized control input. The water-relative velocity $\nu_{r}$ is defined in Section 2.2.

### 2.2 Flow-field representation

Let $D\subset R^{2}$ denote the two-dimensional North–East flow-map domain. A generic point in this domain is presented as $q={\left[\begin{array}{cc} x_{q} & y_{q} \end{array}\right]}^{⊤}\in D.$ The horizontal flow velocity expressed in the inertial frame at time t is denoted by

$$v_{f}^{n}\left(q,t\right)=\left[\begin{array}{c} u_{f}\left(q,t\right)\\ v_{f}\left(q,t\right) \end{array}\right],$$

where $u_{f}$ and $v_{f}$ are the North and East components of the flow, respectively. At the vehicle position p(t) , the inertial-frame flow is transformed into the body frame as

$$v_{f}^{b}\left(t\right)=R_{n}^{b}\left(\psi \right)\left[\begin{array}{c} v_{f}^{n}\left(p\left(t\right),t\right)\\ 0 \end{array}\right].$$

The water-relative velocity is

$$\nu_{r}=\nu -v_{f}^{b},$$

where $v_{f}^{b}$ is the local flow velocity expressed in the body frame.

The flow is not assumed to be globally constant. Instead, it is represented by a grid-based flow map over D . Each map cell m stores a local horizontal flow estimate ${\hat{v}}_{f,m}^{\;n}$ and covariance $\Sigma_{m}^{\text{flow}}$ . Interpolation over the grid provides the estimated flow ${\hat{v}}_{f}^{\;n}\left(q,t\right)$ and covariance $\Sigma^{\text{flow}}\left(q,t\right)$ at vehicle and patch-boundary locations. In the EKF, only the flow estimate in the active map cell m(t) containing the vehicle is included in the state, while the full map is stored and updated cell-wise. Map cells that are not currently active retain their stored flow estimates and covariances. However, their uncertainty is allowed to increase with time until they are revisited or corrected by boundary-derived pseudo-measurements. As a result, patches propagating through stale or poorly observed map regions inherit larger flow uncertainty, which appears as increased boundary uncertainty.

### 2.3 Patch representation

At time t , the vehicle maintains an active set of patch indices, I(t) , and the number of active patches is

$$N_{p}\left(t\right)=|I\left(t\right)|.$$

The set I(t) contains the indices of the patches currently maintained by the onboard tracking and scheduling framework. The scalar $N_{p}\left(t\right)$ is, therefore, the number of active patch tracks, not a patch state. For each active patch i∈I(t) , the physical patch area is modeled as a bounded two-dimensional region:

$$A_{i}\left(t\right)\subset D.$$

The region $A_{i}\left(t\right)$ represents the area occupied by the environmental feature, such as a plume, algal bloom, oil slick, or floating-debris patch. The true patch region is not assumed to be convex. In computation, the boundary of the physical patch is represented by a polygonal approximation:

$$B_{i}\left(t\right)={\left\{b_{ij}\left(t\right)\right\}}_{j=1}^{N_{B}},$$

where $b_{ij}\left(t\right)\in R^{2}$ is the j th ordered boundary vertex and $N_{B}$ is the fixed number of vertices used after resampling. The polygon $B_{i}\left(t\right)$ approximates the true outer contour of the physical patch area $A_{i}\left(t\right)$ . The vertices are ordered counterclockwise when the boundary is simple. Each boundary vertex has an associated covariance $\Sigma_{ij}^{\text{bdry}}\left(t\right)\in R^{2\times 2}$ , which represents uncertainty in the estimated position of vertex j of patch i . The scalar uncertainty of patch i is defined as follows:

$$J_{i}\left(t\right)=\overset{N_{B}}{\underset{j=1}{\sum }}\text{tr }\;\left(\Sigma_{ij}^{\text{bdry}}\left(t\right)\right).$$

The centroid of the polygonal approximation is denoted by $c_{i}\left(t\right)$ . Since the patch area is not restricted to be convex, the geometric centroid may occasionally lie outside the area or may be a poor service point. Therefore, the centroid is used only when it is admissible; otherwise, the reference-generation module selects an admissible service point $s_{i}\left(t\right)$ on or inside the regularized boundary. The admissible service point is recomputed from the current regularized boundary whenever the patch geometry is updated; it is not treated as a fixed point attached to the initial patch.

The active set I(t) may change during the mission due to patch merge or split events. However, because a single vehicle cannot maintain an unbounded number of patch tracks, the onboard active set is capped by

$$N_{p}\left(t\right)\leq N_{\max},$$

where $N_{\max}$ is the maximum number of patches maintained by the onboard tracking and scheduling framework.

Table 1 summarizes the main notations used throughout the paper.

**TABLE 1:** Main notations used in the paper.

Symbol	Description
Vehicle and flow	
D	Two-dimensional North–East flow-map domain
$\eta ={\left[x,y,\psi \right]}^{⊤}$	Vehicle pose in the inertial frame
$\nu ={\left[u,v,r\right]}^{⊤}$	Body-frame surge, sway, and yaw-rate vector
$\nu_{r}$	Water-relative body-frame velocity
$R_{b}^{n}\left(\psi \right),R_{n}^{b}\left(\psi \right)$	Body-to-inertial and inertial-to-body rotations
p(t)	Vehicle horizontal position
$v_{f}^{n}\left(q,t\right)$	Flow velocity at spatial point q , expressed in the inertial frame
${\hat{v}}_{f}^{\;n}\left(q,t\right)$	Interpolated estimated flow velocity at point q
$\Sigma^{\text{flow}}\left(q,t\right)$	Interpolated flow-map covariance at point q
$x_{E}$	Augmented EKF state for vehicle motion, local flow, and biases
Patch representation and uncertainty	
I(t)	Active set of candidate patch indices maintained onboard
$N_{p}\left(t\right)$	Number of active patches, $N_{p}\left(t\right)=|I\left(t\right)|$
$N_{\max}$	Maximum number of onboard patch tracks
$A_{i}\left(t\right)$	Physical area occupied by patch i
$B_{i}\left(t\right)$	Polygonal approximation of the outer contour of $A_{i}\left(t\right)$
$b_{ij}\left(t\right)$	Boundary vertex j of patch i
$N_{B}$	Number of vertices per patch after resampling
$\Sigma_{ij}^{\text{bdry}}\left(t\right)$	Covariance of boundary vertex j of patch i
$J_{i}\left(t\right)$	Scalar uncertainty metric of patch i
$c_{i}\left(t\right)$	Geometric centroid of patch i
$s_{i}\left(t\right)$	Admissible service point when the centroid is unsuitable
$r_{i}\left(t\right)$	Patch-level reference/service point associated with patch i
r(t)	Tracking reference sent to the controller
R(⋅)	Boundary regularization/resampling operator
Scheduling and mission timing	
$R_{\text{visit}}$	Visit-region radius used to initiate a patch service event
$T_{\text{sch}}$	Scheduler update period
$T_{i}$	Predicted travel time to patch i
$E_{i}$	Predicted travel-effort proxy for patch i
$T_{\text{svc}}$	Nominal dwell time required to complete a patch service update
$T_{\max}$	Maximum desired revisit interval
$T_{i}^{\text{age}}\left(t\right)$	Time since patch i was last serviced
$\Phi_{i}$	Scheduler score for patch i
$i^{⋆}$	Selected patch index
L(S)	Closed-tour length through patch set S
$v_{\text{eff}}$	Conservative lower bound on achievable ground speed

### 2.4 Initialization and deployment assumptions

#### 2.4.1 Assumption 1: initial candidate patches

At the beginning of the mission, an external detection (satellite imagery and aerial) or survey stage (drone-based surveys, shore-based sensing, and a prior vehicle survey) provides an initial active set of candidate patch indices:

$$I\left(0\right)=\left\{1,\ldots ,N_{p}\left(0\right)\right\},$$

and corresponding polygonal boundary approximations:

$${\left\{B_{i}\left(0\right)\right\}}_{i\in I\left(0\right)}.$$

The initial boundaries are not assumed to be exact. Their uncertainty is encoded through the initial vertex covariances ${\left\{\Sigma_{ij}^{\text{bdry}}\left(0\right)\right\}}_{j=1}^{N_{B}}.$ Thus, the framework starts from uncertain candidate patch boundaries rather than from perfectly known patch geometries.

#### 2.4.2 Assumption 2: initial flow prior

The initial flow map may be obtained from shore-based HF radar, an ocean or coastal forecast product, prior surveys, or drifter/current-meter measurements. In this study, the HF-radar replay provides the flow field used for validation. In deployment, the external flow product provides a prior map that is locally refined by the onboard estimator and by service-event boundary updates.

#### 2.4.3 Assumption 3: candidate-set limitations

The number and location of the initial candidate patches affect the quality of the subsequent tracking mission. False-positive candidates may be removed or merged during patch-set m\nagement, and split events may create additional candidate patches. However, patches that are completely missed by the initialization stage and never enter the vehicle’s sensing range are outside the scope of the present formulation. Such new-patch discovery requires either an external detection update or an exploration layer, which is left for future work.

#### 2.4.4 Assumption 4: service-event abstraction

A service event is modeled as the acquisition of noisy boundary measurements for the selected patch within the vehicle’s local sensing range. These measurements may come from a camera, sonar, fluorometer-informed contour extraction, or another task-specific boundary detector. The vehicle is not assumed to observe the true patch boundary perfectly. Instead, the detected boundary points or local contour segments are treated as noisy measurements that are associated with the predicted polygonal boundary using a geometric association step, such as nearest-boundary point matching or point-to-segment projection along the predicted contour. The associated measurements are then used to correct the corresponding boundary-vertex estimates.

#### 2.4.5 Assumption 5: onboard execution

The proposed framework is intended to run onboard the vehicle during deployment. External resources (ocean models, maps, etc.) are used for initialization and mission planning, but the closed-loop operation does not require continuous cloud connectivity or remote closed-loop control.

These assumptions define the scope of this study. Now, even when patches are initially detected from satellite or aerial imagery, persistent vehicle tracking remains useful because the initial detection is only a snapshot. Surface patches can translate, deform, stretch, split, merge, or become partially obscured under spatially varying flow. The vehicle, therefore, provides local, repeated boundary reacquisition and uncertainty reduction between external remote-sensing updates.

### 2.5 Problem statement

Under the modeling setup and deployment assumptions stated above, the proposed framework addresses a coupled estimation, propagation, scheduling, and control problem. It can be represented mathematically by a decision set:

$$\left\{i^{⋆}\left(t\right),\;r\left(t\right),\;u_{\text{cmd}}\left(t\right)\right\},$$

where $i^{⋆}\left(t\right)\in I\left(t\right)$ is the index of the patch to be tracked at any given time, $r\left(t\right)\in R^{2}$ is the reference generated from the selected patch geometry (for example, patch centroid), and $u_{\text{cmd}}\left(t\right)={\left[\begin{array}{cc} a\left(t\right) & \omega \left(t\right) \end{array}\right]}^{⊤}$ is the vehicle command, with a(t) denoting the commanded surge acceleration and ω(t) denoting the commanded yaw rate.

Before stating the persistent tracking problem, we define two operational quantities used throughout the paper. The first specifies when a patch visit produces a service update, and the second specifies what it means for patch uncertainty to remain controlled during a mission.

Definition 1 *Service event. For patch* i *, let* $r_{i}\left(t\right)$ *denote the reference point associated with that patch. A service event is initiated when the vehicle enters the prescribed visit region:*

$$\|p\left(t\right)-r_{i}\left(t\right)\|\leq R_{\text{visit}},$$

where p(t) is the vehicle position and $R_{\text{visit}}$ is the visit radius. The patch is considered serviced after the vehicle remains within this visit region for a nominal service duration, $T_{\text{svc}}$ , or equivalently, after sufficient local boundary measurements have been acquired.

Definition 2 *Boundedness of patch uncertainty. For each active patch* i∈I(t) *, the scalar boundary uncertainty is*

$$J_{i}\left(t\right)=\overset{N_{B}}{\underset{j=1}{\sum }}\text{tr }\left(\Sigma_{ij}^{\text{bdry}}\left(t\right)\right).$$

The patch uncertainties are said to remain practically bounded over a mission horizon [0,T] if there exists a finite threshold $J_{\max}$ such that

$$J_{i}\left(t\right)\leq J_{\max},\;\forall i\in I\left(t\right),\;t\in \left[0,T\right].$$

The overall problem is to design an onboard closed-loop framework that computes $\left\{i^{⋆}\left(t\right),\;r\left(t\right),\;u_{\text{cmd}}\left(t\right)\right\}$ , so that the uncertainty metrics $J_{i}\left(t\right)$ of all maintained patches remain practically bounded during persistent operation, while unnecessary travel and control effort are reduced. This problem can be effectively decoupled into three distinct sub-problems.

Problem #1. *Given the vehicle measurements, an initial flow-map prior, and the active patch set* I(t) *, estimate the vehicle state, local flow, and propagate the patch boundary.*

Patch propagation involves propagating the patch boundary approximation $B_{i}\left(t\right)$ and the set of vertex covariance ${\left\{\Sigma_{ij}^{\text{bdry}}\left(t\right)\right\}}_{j=1}^{N_{B}}$ under the estimated flow field.

Problem #2. *Given the active patch set* I(t) *, the uncertainty metrics* $\left\{J_{i}\left(t\right)\right\}$ *, the predicted travel times* $\left\{T_{i}\right\}$ *, and the predicted travel-effort proxies* $\left\{E_{i}\right\}$ *, select the index of the patch*

$$i^{⋆}\left(t\right)\in I\left(t\right)$$

to be serviced.

The scheduling policy is implemented as a receding-horizon urgency-ranking strategy based on predicted uncertainty growth and flow-aware travel-effort estimates.

Problem #3. *Given the selected patch* $i^{⋆}\left(t\right)$ *, compute a tracking reference* r(t) *from the selected patch geometry and generate the vehicle command* $u_{\text{cmd}}\left(t\right)$ *so that the vehicle reaches and services the selected patch while accounting for the estimated surrounding flow.*

## 3 Technical methods

This section outlines the onboard estimation, patch propagation, boundary fusion, reference generation, tracking control, and patch-selection modules employed to address Problems #1, #2, and #3. The proposed framework is structured as a multi-rate closed-loop architecture. We denote continuous mission time by t . The fast onboard loop is indexed by k , with

$$t_{k}=k\Delta t,$$

where Δt is the estimation-control time step. The EKF, patch propagation, reference generation, and tracking controller are updated on this fast time grid. The mission scheduler is updated less frequently, every $T_{\text{sch}}$ seconds, or equivalently every

$$K_{\text{sch}}=\frac{T_{\text{sch}}}{\Delta t}$$

fast-loop samples when $T_{\text{sch}}$ is an integer multiple of Δt . This multi-rate structure reflects the operational separation between fast vehicle-level estimation and control and slower mission-level patch-selection decisions.

At each fast-loop cycle, the vehicle updates its state and the local flow estimate using onboard sensor measurements. The active patch boundaries are then propagated through the estimated flow map, their boundary uncertainties are updated, and a tracking reference is generated from the selected patch geometry. The vehicle applies a flow-aware gain-scheduled LQR controller to track this reference. At the slower mission level, the scheduler evaluates the active patches using predicted arrival uncertainty, revisit age, and travel effort cost and selects the next patch to service. When a service update is completed, boundary measurements are fused with the selected patch boundary, and nearby flow-map covariances may be refined using boundary-derived pseudo-measurements.

**FIGURE 1:**
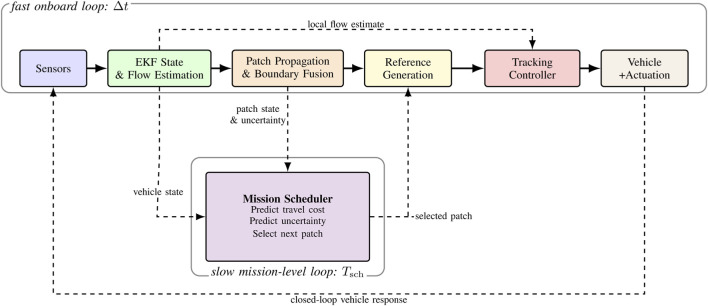
System block diagram showing the fast onboard estimation–propagation–tracking loop and the slower mission-level multi-patch scheduler. Solid arrows indicate the main forward processing chain, while dashed arrows indicate auxiliary feedback and scheduler-level information flow.

All modules use onboard-available quantities: the current vehicle state estimate, the stored flow-map estimate and covariance, the stored patch-boundary states, and intermittent boundary measurements. The overall architecture is shown in Figure 1, and the closed-loop workflow is summarized in Algorithm 1.

Algorithm 1 **High-level uncertainty-aware multi-patch tracking loop.**

1: Initialize vehicle state estimate, flow-map prior, active patch set I(0) , and patch-boundary states
2: **for** each fast-loop time step **do**
3:  Update the vehicle state and active-cell flow estimate using the EKF
4:  Propagate active patch boundaries through the estimated flow map
5:  Propagate boundary covariances and update the patch uncertainties
6:  **if** a scheduler update is due **then**
7:   Predict travel time, travel effort, and arrival uncertainty for reachable patches
8:   Select the next patch $i^{⋆}$ using the scheduling score
9:  **end if**
10:   Generate the tracking reference from the selected patch geometry
11:   Apply the flow-aware gain-scheduled LQR tracking controller
12:   **if** a service update is completed for the selected patch **then**
13:   Fuse boundary measurements into the selected patch boundary
14:   Refine nearby flow-map covariances when boundary-derived measurements are available
15:   Update the active patch set after any split or merge event
16:   Reset the revisit age of the serviced patch
17:  **end if**
18: **end for**

### 3.1 Flow estimation

The vehicle state and local flow are estimated using an EKF. The local flow is not measured directly; instead, it is inferred from the prediction–measurement mismatch, or innovation, between the vehicle dynamic model and the bottom-track Doppler velocity log (DVL) ground-velocity measurements. Therefore, reliable flow estimation requires sufficient vehicle excitation, a usable bottom-track DVL signal, and a nominal vehicle model that is accurate over the operating envelope.

The EKF uses the augmented state

$$x_{E}={\left[\begin{array}{ccccc} \eta^{⊤} & \nu^{⊤} & {\left(v_{f,m\left(k\right)}^{n}\right)}^{⊤} & b_{r} & b_{\psi } \end{array}\right]}^{⊤}\in R^{10}.$$

The pose η and body-frame velocity ν are defined in Section 2. The vector

$$v_{f,m\left(k\right)}^{n}={\left[\begin{array}{cc} u_{f,m\left(k\right)} & v_{f,m\left(k\right)} \end{array}\right]}^{⊤}$$

denotes the horizontal flow state associated with the active map cell containing the vehicle, while $b_{r}$ and $b_{\psi }$ denote gyro and heading biases, respectively. The full flow map is stored cell-wise, but only the active-cell flow state is included in the EKF at a given time.

The process model uses the planar vehicle dynamics introduced in Section 2. In compact form, we can obtain Equation 1:

$${\dot{x}}_{E}=f_{E}\left(x_{E},\tau \right)+w_{E},\;w_{E}∼N\left(0,Q_{E}\right).$$ (1)

Equivalently, the vehicle pose and velocity evolve according to the 3-DOF vehicle model, while the active-cell flow and bias states are modeled as random walks:

$${\dot{v}}_{f,m\left(k\right)}^{n}=w_{f},\;{\dot{b}}_{r}=w_{b_{r}},\;{\dot{b}}_{\psi }=w_{b_{\psi }}.$$

The augmented EKF state combines fast vehicle states with more slowly varying flow and bias states. The vehicle pose and body-frame velocity evolve through the controlled 3-DOF dynamics, while the active-cell flow and sensor biases are modeled as random walks. The corresponding process-noise covariances determine how quickly the EKF allows these quantities to vary. This stochastic representation is used only to support joint vehicle/flow estimation; it is not intended as a formal singular-perturbation model.

The measurement vector contains heading, yaw-rate, and bottom-track DVL ground-velocity measurements:

$$z_{k}={\left[\begin{array}{cccc} \psi_{\text{mag},k} & r_{\text{gyro},k} & v_{x_{\left(DVL\right)},k}^{n} & v_{y_{\left(DVL\right)},k}^{n} \end{array}\right]}^{⊤}.$$

The corresponding measurement model is presented in Equation 2:

$$z_{k}=h\left(x_{E,k}\right)+\epsilon_{k},\;\epsilon_{k}∼N\left(0,\Sigma_{z}\right),$$ (2)

with

$$h\left(x_{E}\right)=\left[\begin{array}{c} \psi +b_{\psi }\\ r+b_{r}\\ SR_{b}^{n}\left(\psi \right)\nu \end{array}\right],\;S=\left[\begin{array}{ccc} 1 & 0 & 0\\ 0 & 1 & 0 \end{array}\right].$$

Here, the DVL is modeled in bottom-track mode, so it measures vehicle ground velocity rather than water-relative velocity. The present formulation assumes bottom-track DVL availability during the reported experiments. Handling prolonged bottom-lock loss is outside the scope of this study.

The flow state affects the EKF prediction through the water-relative velocity

$$\nu_{r}=\nu -v_{f}^{b},$$

where $v_{f}^{b}$ is the local flow expressed in the body frame. Thus, the local flow is estimated indirectly through consistency between the vehicle model and the heading, yaw-rate, and DVL measurements.

At each time step, the EKF prediction is computed as shown in Equations 3, 4:

$${\hat{x}}_{E,k|k-1}={\hat{x}}_{E,k-1|k-1}+\Delta t\;f_{E}\left({\hat{x}}_{E,k-1|k-1},\tau_{k-1}\right),$$ (3)

$$P_{E,k|k-1}=F_{E,k}P_{E,k-1|k-1}F_{E,k}^{⊤}+Q_{E,k},$$ (4)

where

$$F_{E,k}={\left.\frac{\partial }{\partial x_{E}}\left(x_{E}+\Delta tf_{E}\left(x_{E},\tau_{k-1}\right)\right)\right|}_{{\hat{x}}_{E,k-1|k-1}}.$$

The correction step uses the standard EKF measurement update with the linearized measurement Jacobian

$$H_{E,k}={\left.\frac{\partial h}{\partial x_{E}}\right|}_{{\hat{x}}_{E,k|k-1}}.$$

The full Jacobian follows directly from the compact measurement model above and is not expanded here to keep the notation concise.

The flow map is maintained cell-wise. When the vehicle enters a new map cell, the EKF stores the updated active-cell flow estimate and covariance back into the map; only the local flow component of the EKF state is replaced by the stored estimate for the new active cell. To avoid overconfidence after a cell transition, the loaded flow covariance is inflated before the next update. This allows the EKF to adapt to the newly loaded local flow estimate without introducing a strong transient caused by an overconfident cell switch.

Map cells that are not currently active retain their stored flow estimates and covariances. For cells that are not revisited or corrected by boundary-derived pseudo-measurements (detailed in Section 3.3), the stored covariance is allowed to age, so patches propagated through stale regions naturally accumulate larger boundary uncertainty. The cell-wise EKF flow update is summarized in Algorithm 2.

Algorithm 2 **Cell-wise EKF flow update at the fast-loop sample *k*.**

**Require:** Control input $\tau_{k-1}$ , measurement $z_{k}$ , and active map cell m(k)

**Ensure:** Updated EKF estimate ${\hat{x}}_{E,k|k}$ , covariance $P_{E,k|k}$ , and cell-wise flow-map entry
1: **if** active map cell has changed **then**
2:  Store the previous active-cell flow estimate and covariance in the flow map
3:  Load the stored flow estimate and covariance for the new active cell
4:  Replace only the active-cell flow component in ${\hat{x}}_{E,k-1|k-1}$

5:  Inflate the loaded flow covariance to reduce overconfidence after the cell transition
6: **end if**
7: **Prediction**
8: Compute the body-frame flow $v_{f}^{b}$ and water-relative velocity $\nu_{r}$

9: Predict the augmented EKF state:

$${\hat{x}}_{E,k|k-1}\leftarrow {\hat{x}}_{E,k-1|k-1}+\Delta t\;f_{E}\left({\hat{x}}_{E,k-1|k-1},\tau_{k-1}\right)$$

10: Linearize the discrete prediction model to obtain $F_{E,k}$

11: Propagate the EKF covariance:

$$P_{E,k|k-1}\leftarrow F_{E,k}P_{E,k-1|k-1}F_{E,k}^{⊤}+Q_{E,k}$$

12: Wrap the yaw estimate ψ to the chosen angle interval
13: **Measurement update**
14: Update the yaw-rate state using the gyro measurement
15: Update the heading state using the magnetometer measurement
16: Update the ground-velocity components using the bottom-track DVL measurement
17: Re-symmetrize the EKF covariance:

$$P_{E,k|k}\leftarrow \frac{1}{2}\left(P_{E,k|k}+P_{E,k|k}^{⊤}\right)$$

18: **Map storage**
19: Store the updated active-cell flow estimate and covariance back into map cell m(k)

20: Allow non-active map-cell covariances to age until revisited or corrected by boundary-derived measurements (detailed Section 3.3)

### 3.2 Patch propagation and reference-point generation

**FIGURE 2:**
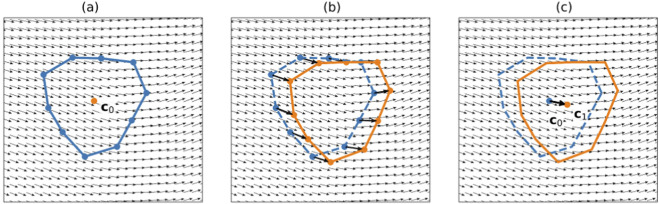
Illustration of patch-boundary propagation under spatially varying flow. **(a)** Initial patch. **(b)** Vertex-wise advection. **(c)** Updated patch and centroid shift.

The patch-tracking problem has a mixed continuous–discrete character. Between service events, each patch boundary is propagated through the estimated flow field, and the corresponding boundary covariance grows according to local flow uncertainty. When the vehicle services a patch, boundary measurements become available at discrete event times. At those service events, the predicted boundary is corrected using the service-event boundary-fusion update described in Subsection 3.3. Thus, each patch evolves by flow-driven advection between visits and by measurement-driven correction at service events. Figure 2 illustrates the vertex-wise boundary propagation process under a spatially varying flow field.

Let

$${\hat{v}}_{f}^{\;n}\left(q,t\right)=\left[\begin{array}{c} {\hat{u}}_{f}\left(q,t\right)\\ {\hat{v}}_{f}\left(q,t\right) \end{array}\right]$$

denote the interpolated estimated flow velocity at a spatial point q∈D . For patch i , the j th boundary vertex $b_{ij}\left(t\right)$ evolves according to Equation 5:

$${\dot{b}}_{ij}={\hat{v}}_{f}^{\;n}\left(b_{ij},t\right).$$ (5)

Using a discrete propagation step Δt , the nominal vertex update is presented in Equation 6:

$$b_{ij}^{+}=b_{ij}+\Delta t\;{\dot{b}}_{ij}.$$ (6)

The centroid is not propagated as an independent state. Instead, it is recomputed from the advected boundary vertices. This allows the reference motion to reflect the collective deformation of the patch boundary under spatially varying flow.

Because the interpolated flow map is uncertain, the stochastic vertex propagation is modeled as Equation 7:

$$b_{ij}^{+}=b_{ij}+\Delta t\;{\dot{b}}_{ij}+\Delta t\;\xi_{ij}^{\text{flow}}+\xi_{ij}^{\text{adv}},$$ (7)

where

$$\xi_{ij}^{\text{flow}}∼N\;\left(0,\Sigma^{\text{flow}}\left(b_{ij},t\right)\right),\;\xi_{ij}^{\text{adv}}∼N\left(0,\Sigma^{\text{adv}}\right).$$

Here, $\Sigma^{\text{flow}}\left(b_{ij},t\right)$ is the interpolated flow-map velocity covariance at the vertex location, while $\Sigma^{\text{adv}}$ is a per-step boundary-process covariance that captures unresolved deformation, numerical integration error, and subgrid-scale advection effects. For computational scalability, cross-covariances between distinct boundary vertices are neglected, so each boundary-vertex covariance is propagated independently. The propagation model treats the prior boundary uncertainty, flow-map uncertainty, and unresolved advection disturbance as mutually independent.

A covariance propagation is used for each boundary vertex by linearizing the local flow map about the current vertex position. Let

$$F_{ij}=I_{2}+\Delta t{\left.\frac{\partial {\hat{v}}_{f}^{\;n}}{\partial q}\right|}_{q=b_{ij}}$$

denote the local discrete-time propagation Jacobian. This Jacobian captures the local spatial variation of the interpolated flow around the current boundary vertex. The boundary-vertex covariance is then propagated as Equation 8:

$$\Sigma_{ij}^{\text{bdry},+}=F_{ij}\Sigma_{ij}^{\text{bdry}}F_{ij}^{⊤}+\Delta t^{2}\Sigma^{\text{flow}}\left(b_{ij},t\right)+\Sigma^{\text{adv}}.$$ (8)

The term $\Delta t^{2}\Sigma^{\text{flow}}\left(b_{ij},t\right)$ accounts for uncertainty in the interpolated flow velocity integrated over one propagation step, while $\Sigma^{\text{adv}}$ accounts for additional per-step boundary process uncertainty.

After advection, a lightweight boundary regularization operator R(⋅) is applied to keep a fixed number of approximately uniformly spaced boundary vertices. In practice,

$$\left({\hat{B}}_{i},{\left\{{\hat{\Sigma }}_{ij}^{\text{bdry}}\right\}}_{j=1}^{N_{B}}\right)=R\;\left(B_{i}^{+},{\left\{\Sigma_{ij}^{\text{bdry},+}\right\}}_{j=1}^{N_{B}},N_{B}\right).$$

Here, $B_{i}^{+}$ denotes the advected boundary before regularization, while ${\hat{B}}_{i}$ denotes the regularized boundary used for subsequent reference generation and service-event updates. In practice, R(⋅) redistributes vertices along the advected contour using arc-length resampling. The covariance associated with a new vertex is obtained from the nearest propagated vertex or by local interpolation of neighboring propagated covariances.

After advection and regularization, the centroid of patch i is computed as follows:

$$c_{i}\left(t\right)=\text{centroid }\;\left({\hat{B}}_{i}\left(t\right)\right).$$

Because no convexity assumption is imposed on the patch area, the geometric centroid is not always guaranteed to be an admissible service point. Therefore, the patch service point is selected as follows:

$$r_{i}\left(t\right)=\left\{\begin{array}{cc} c_{i}\left(t\right), & \text{if }c_{i}\left(t\right)\text{ is admissible},\\ s_{i}\left(t\right), & \text{otherwise}, \end{array}\right.$$

where $s_{i}\left(t\right)$ is an admissible service point on or inside the regularized patch boundary. An admissible service point is a point that is reachable by the vehicle and suitable for local sensing and boundary reacquisition. Both $c_{i}\left(t\right)$ and $s_{i}\left(t\right)$ are recomputed whenever the patch geometry is updated, including after advection, regularization, service-event correction, and scheduler updates.

Algorithm 3 summarizes the single-patch update used by the onboard framework. The same procedure is applied to each active patch i∈I(t) , while the scheduler described in Section 3.3 selects which patch is serviced next.

Algorithm 3 **Patch propagation, service update, and reference generation for patch i.**

**Require:** Patch boundary $B_{i}={\left\{b_{ij}\right\}}_{j=1}^{N_{B}}$ and boundary covariances ${\left\{\Sigma_{ij}^{\text{bdry}}\right\}}_{j=1}^{N_{B}}$

**Require:** Estimated flow map ${\hat{v}}_{f}^{\;n}\left(\cdot ,t\right)$ , flow covariance $\Sigma^{\text{flow}}\left(\cdot ,t\right)$ , and step size Δt

**Require:** Optional detected boundary ${\tilde{B}}_{i}$ from a completed service event
**Ensure:** Regularized boundary ${\hat{B}}_{i}$ , updated covariances, patch reference $r_{i}$ , and uncertainty $J_{i}$

1: **Prediction: boundary advection and covariance propagation**
2: **for** j←1 to $N_{B}$ **do**
3:  Advect vertex $b_{ij}$ using the estimated flow ${\hat{v}}_{f}^{\;n}\left(b_{ij},t\right)$

4:  Compute the local propagation Jacobian $F_{ij}$

5:  Propagate $\Sigma_{ij}^{\text{bdry}}$ using (8)
6:  Symmetrize $\Sigma_{ij}^{\text{bdry}}$

7: **end for**
8: Form the advected boundary $B_{i}^{+}$

9: $\left({\hat{B}}_{i},\left\{{\hat{\Sigma }}_{ij}^{\text{bdry}}\right\}\right)\leftarrow R\left(B_{i}^{+},\left\{\Sigma_{ij}^{\text{bdry}}\right\},N_{B}\right)$

10: **Service-event boundary update**
11: **if** ${\tilde{B}}_{i}$ is available **then**
12:  Resample ${\tilde{B}}_{i}$ to $N_{B}$ boundary points
13:  Register ${\tilde{B}}_{i}$ to ${\hat{B}}_{i}$ using contour alignment
14:  Associate detected points to predicted vertices by nearest arc-length correspondence
15:  **for** j←1 to $N_{B}$ **do**
16:  Apply the boundary-fusion update to $b_{ij}$ and $\Sigma_{ij}^{\text{bdry}}$

17:  Symmetrize $\Sigma_{ij}^{\text{bdry}}$

18:  **end for**
19:  Regularize the corrected boundary using R(⋅)

20:  **if** successive associated detections are available **then**
21:   Form boundary-displacement pseudo-measurements
22:   Refine nearby flow-map cells and their covariances
23:  **end if**
24: **end if**
25: **Reference-point generation**
26: $c_{i}\leftarrow \text{centroid }\left({\hat{B}}_{i}\right)$

27: **if** $c_{i}$ is admissible **then**
28: $r_{i}\leftarrow c_{i}$

29: **else**
30: $s_{i}\leftarrow \text{NearestAdmissiblePoint }\left({\hat{B}}_{i},c_{i}\right)$

31: $r_{i}\leftarrow s_{i}$

32: **end if**
33: **Patch uncertainty**
34: $J_{i}\leftarrow \sum_{j=1}^{N_{B}}\text{tr }\;\left(\Sigma_{ij}^{\text{bdry}}\right)$

35: **return** ${\hat{B}}_{i},{\left\{\Sigma_{ij}^{\text{bdry}}\right\}}_{j=1}^{N_{B}},c_{i},s_{i},r_{i},J_{i}$

### 3.3 Patch service update and multi-patch scheduling

The active patch set I(t) , the number of active patches $N_{p}\left(t\right)$ , and the onboard capacity $N_{\max}$ were defined in Section 2.3. For each active patch i∈I(t) , the framework stores the polygonal boundary approximation $B_{i}\left(t\right)$ , the centroid $c_{i}\left(t\right)$ , the boundary-vertex covariances ${\left\{\Sigma_{ij}^{\text{bdry}}\left(t\right)\right\}}_{j=1}^{N_{B}},$ and the last service time $t_{i}^{\text{last}}$ .

The vehicle services one active patch at a time. Service events follow Definition 1. In the technical implementation, entering the visit region initiates boundary measurement collection for the selected patch, and the service update is completed after the nominal dwell time $T_{\text{svc}}$ or once sufficient boundary measurements have been acquired and fused. Patch selection is performed by a scheduler operating at the slower mission-level period $T_{\text{sch}}$ , while patch propagation and tracking control run at the fast onboard period Δt .

Patch uncertainty and service-event correction: Between service events, each patch boundary is propagated through the estimated flow field using the model in Section 3.2. During this unobserved advection period, boundary uncertainty grows according to the local flow uncertainty and unresolved boundary-process effects. The scalar uncertainty of patch i is presented in Equation 9:

$$J_{i}\left(t\right)=\overset{N_{B}}{\underset{j=1}{\sum }}\text{tr }\left(\Sigma_{ij}^{\text{bdry}}\left(t\right)\right).$$ (9)

Thus, larger $J_{i}\left(t\right)$ indicates lower confidence in the estimated boundary location.

When the vehicle services patch i , boundary observations are obtained from the onboard boundary detector. The detected contour or boundary points are resampled and geometrically aligned with the predicted polygonal boundary to produce associated vertex measurements:

$${\tilde{b}}_{ij}=b_{ij}+\epsilon_{ij}^{\text{det}},\;\epsilon_{ij}^{\text{det}}∼N\left(0,\Sigma^{\text{det}}\right),$$

where $\Sigma^{\text{det}}$ is the boundary-detection covariance. A Kalman-style correction is then applied independently to each associated boundary vertex (Equations 10–13):

$$S_{ij}=\Sigma_{ij}^{\text{bdry}}+\Sigma^{\text{det}},$$ (10)

$$K_{ij}=\Sigma_{ij}^{\text{bdry}}S_{ij}^{-1},$$ (11)

$$b_{ij}\leftarrow b_{ij}+K_{ij}\left({\tilde{b}}_{ij}-b_{ij}\right),$$ (12)

$$\Sigma_{ij}^{\text{bdry}}\leftarrow \left(I_{2}-K_{ij}\right)\Sigma_{ij}^{\text{bdry}}{\left(I_{2}-K_{ij}\right)}^{⊤}+K_{ij}\Sigma^{\text{det}}K_{ij}^{⊤}.$$ (13)

This update reduces the instantaneous boundary uncertainty of the serviced patch.

Successive boundary detections can also provide local flow information. If two associated boundary detections are available over a time interval ΔT , their displacement gives the boundary-derived pseudo-measurement (Equation 14):

$$z_{ij}^{\text{flow}}=\frac{{\tilde{b}}_{ij}\left(t+\Delta T\right)-{\tilde{b}}_{ij}\left(t\right)}{\Delta T}\approx {\hat{v}}_{f}^{\;n}\left(b_{ij},t\right)+\epsilon_{ij}^{\text{flow}}.$$ (14)

These pseudo-measurements are used to locally refine nearby cell-wise flow estimates and reduce the flow-map covariance $\Sigma^{\text{flow}}\left(\cdot ,t\right)$ near the serviced boundary. Consequently, servicing a patch can reduce both the current boundary uncertainty and the future uncertainty growth rate during subsequent propagation.

Patch split, merge, and active-set management: Although each tracked patch is represented using a fixed number of boundary vertices $N_{B}$ , the active patch set itself is not assumed to be topologically fixed. Spatially varying flow can stretch, deform, merge, or separate patch boundaries. Therefore, the active set I(t) is allowed to change during the mission through merge and split events.

If two predicted or observed patch boundaries overlap, or if their separation falls below a prescribed merge tolerance, they are treated as a merged patch. The resulting boundary is regularized using R(⋅) , resampled to $N_{B}$ vertices, and assigned an inflated covariance to account for uncertainty introduced by the topological update.

Conversely, if a serviced boundary observation separates into multiple connected components, the parent patch is split into child patches. Each child boundary is regularized, resampled to $N_{B}$ vertices, and assigned its own boundary covariance. If the number of resulting candidate patches exceeds the onboard capacity $N_{\max}$ , the scheduler retains the highest-priority components according to the same urgency–effort score used for target selection, while lower-priority components are deferred for future external reacquisition.

Travel-effort and arrival-time prediction: At each scheduler update, the vehicle evaluates each reachable candidate patch i∈I(t) . For each candidate, a coarse kinematic transit is simulated from the current vehicle position to the candidate service point under the estimated inertial-frame flow field. The forward simulation terminates when the vehicle reaches the visit radius $R_{\text{visit}}$ or when a maximum prediction horizon is exceeded. If the maximum horizon is exceeded, the patch is treated as temporarily unreachable.

The predicted travel effort is accumulated as Equation 15:

$$E_{i}=\underset{\rho }{\sum }\|\nu_{r,\rho }{\|}^{2}\Delta t_{\text{pred}},$$ (15)

where $\nu_{r,\rho }$ is the water-relative velocity command during the ρ th step of the coarse transit simulation, and $\Delta t_{\text{pred}}$ is the prediction step used in the transit simulation. This quantity is used as a propulsion/control-effort proxy rather than as a full vehicle power model. It does not include onboard processor energy, which is left for future embedded hardware experiments.

Let $T_{i}$ denote the predicted travel time to patch i . The scheduler propagates the patch boundary and its boundary-vertex covariances forward by duration $T_{i}$ to estimate the arrival uncertainty $J_{i}\left(t+T_{i}\right)$ .

Scheduler score and target selection: Candidate patches are ranked using a predictive urgency-per-effort score, as shown in Equation 16:

$$\Phi_{i}=w_{i}\frac{J_{i}\left(t+T_{i}\right)}{E_{i}+\varepsilon },\;\varepsilon >0.$$ (16)

Here, ε prevents division by zero when the predicted travel effort is very small. The urgency weight is presented in Equation 17:

$$w_{i}=1+\lambda \min\;\left(\frac{T_{i}^{\text{age}}\left(t\right)+T_{i}}{T_{\max}},1\right),$$ (17)

where

$$T_{i}^{\text{age}}\left(t\right)=t-t_{i}^{\text{last}}$$

is the time since patch i was last serviced, $T_{\max}$ is the desired maximum revisit interval, and λ>0 controls the strength of the urgency term. Thus, $w_{i}$ increases as the predicted revisit age at arrival approaches the revisit threshold.

**FIGURE 3:**
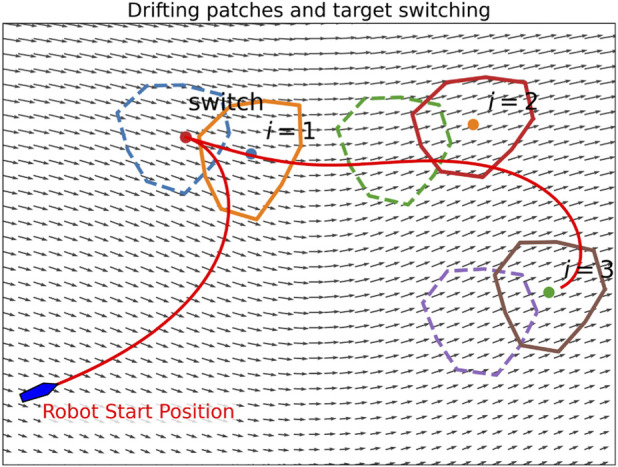
Illustrative multi-patch tracking run.

The selected patch is shown in Equation 18:

$$i^{⋆}=\arg\max_{i\in I_{\text{reach}}\left(t\right)}\Phi_{i},$$ (18)

where $I_{\text{reach}}\left(t\right)\subseteq I\left(t\right)$ is the set of reachable candidate patches. Once a patch is selected, the vehicle remains committed for a minimum service duration. Switching is allowed only if another candidate offers a sufficiently larger score, and a recently serviced patch may be temporarily excluded to encourage coverage of the remaining active set. Figure 3 illustrates a representative multi-patch tracking run with target switching among drifting patches.

Once the scheduler selects $i^{⋆}$ , the tracking reference sent to the controller is the service point of the selected patch,

$$r\left(t\right)=r_{i^{⋆}}\left(t\right),$$

where $r_{i}\left(t\right)$ is computed from the current-regularized patch boundary as described in Section 3.2.

Proposition 1 Bounded patch uncertainty under bounded revisit time.

Consider the boundary-vertex covariance recursion:

$$\Sigma_{n+1}^{\text{bdry}}=F_{n}\Sigma_{n}^{\text{bdry}}F_{n}^{⊤}+\Delta t^{2}\Sigma_{n}^{\text{flow}}+\Sigma^{\text{adv}},$$ (19)

where $\Sigma_{n}^{\text{bdry}}$ , $\Sigma_{n}^{\text{flow}}$ , and $\Sigma^{\text{adv}}$ are symmetric positive semidefinite. Suppose there exist constants $\bar{F}\geq 0$ , $\bar{r}\geq 0$ , and $\bar{q}\geq 0$ such that Equation 20 can be satisfied:

$$\|F_{n}\|\leq \bar{F},\;\text{tr }\;\left(\Sigma_{n}^{\text{flow}}\right)\leq \bar{r},\;\text{tr }\;\left(\Sigma^{\text{adv}}\right)\leq \bar{q}$$ (20)

*for all propagation steps* n . *Here,* $\Sigma_{n}^{\text{flow}}$ *denotes the local flow-map covariance evaluated along the propagated vertex trajectory.*

Let n=0 denote the propagation step immediately after a service-event correction. Assume that each maintained patch is revisited at least once every $K_{\max}$ discrete propagation steps. At each revisit, every boundary vertex receives a boundary measurement update, and the post-update covariance is uniformly bounded by a finite matrix ${\bar{\Sigma }}^{\text{upd}}≻0$ , such that Equation 21 can be satisfied:

$$\Sigma_{0}^{\text{bdry}}⪯{\bar{\Sigma }}^{\text{upd}}.$$ (21)

Let $n^{⋆}$ denote the number of propagation steps between two consecutive visits to the patch, with $n^{⋆}\leq K_{\max}$ . Then, we can obtain (Equation 22):

$$\text{tr }\;\left(\Sigma_{n^{⋆}}^{\text{bdry}}\right)\leq {\bar{F}}^{2n^{⋆}}\text{tr }\;\left(\Sigma_{0}^{\text{bdry}}\right)+\left(\Delta t^{2}\bar{r}+\bar{q}\right)\overset{n^{⋆}-1}{\underset{s=0}{\sum }}{\bar{F}}^{2s}.$$ (22)

Therefore, if the patch uncertainty is defined as Equation 23:

$$J_{i}\left(t\right)=\overset{N_{B}}{\underset{j=1}{\sum }}\text{tr }\left(\Sigma_{ij}^{\text{bdry}}\left(t\right)\right),$$ (23)

then, $J_{i}\left(t\right)$ is uniformly bounded for all maintained patches between service events, with Equation 24:

$$J_{i}\left(t\right)\leq N_{B}\;\bar{p}\left(K_{\max}\right),$$ (24)

where (Equation 25)

$$\begin{array}{cc} \bar{p}\left(K_{\max}\right) & =\alpha^{K_{\max}}\text{tr }\;\left({\bar{\Sigma }}^{\text{upd}}\right)\\ & \;+\left(\Delta t^{2}\bar{r}+\bar{q}\right)\overset{K_{\max}-1}{\underset{s=0}{\sum }}\alpha^{s},\;\alpha =\max\left\{1,{\bar{F}}^{2}\right\}. \end{array}$$ (25)

If α≠1 , then Equation 26 is obtained:

$$\bar{p}\left(K_{\max}\right)=\alpha^{K_{\max}}\text{tr }\;\left({\bar{\Sigma }}^{\text{upd}}\right)+\left(\Delta t^{2}\bar{r}+\bar{q}\right)\frac{1-\alpha^{K_{\max}}}{1-\alpha }.$$ (26)

If α=1 , then Equation 27 can be obtained:

$$\bar{p}\left(K_{\max}\right)=\text{tr }\;\left({\bar{\Sigma }}^{\text{upd}}\right)+K_{\max}\left(\Delta t^{2}\bar{r}+\bar{q}\right).$$ (27)

Proof. Taking the trace of Equation 19 gives Equation 28:

$$\text{tr }\;\left(\Sigma_{n+1}^{\text{bdry}}\right)=\text{tr }\;\left(F_{n}\Sigma_{n}^{\text{bdry}}F_{n}^{⊤}\right)+\Delta t^{2}\text{tr }\;\left(\Sigma_{n}^{\text{flow}}\right)+\text{tr }\;\left(\Sigma^{\text{adv}}\right).$$ (28)

Since $\Sigma_{n}^{\text{bdry}}⪰0$ , Equation 29 is obtained:

$$\begin{array}{ll} \text{tr }\;\left(F_{n}\Sigma_{n}^{\text{bdry}}F_{n}^{⊤}\right) & =\text{tr }\;\left(F_{n}^{⊤}F_{n}\Sigma_{n}^{\text{bdry}}\right)\\ & \;\leq \|F_{n}^{⊤}F_{n}\|\text{tr }\;\left(\Sigma_{n}^{\text{bdry}}\right)\\ & =\|F_{n}{\|}^{2}\text{tr }\;\left(\Sigma_{n}^{\text{bdry}}\right)\\ & \;\leq {\bar{F}}^{2}\text{tr }\;\left(\Sigma_{n}^{\text{bdry}}\right). \end{array}$$ (29)

Therefore, we can obtain Equation 30:

$$\text{tr }\;\left(\Sigma_{n+1}^{\text{bdry}}\right)\leq {\bar{F}}^{2}\text{tr }\;\left(\Sigma_{n}^{\text{bdry}}\right)+\Delta t^{2}\bar{r}+\bar{q}.$$ (30)

Iterating this scalar recursion over a revisit interval of length $n^{⋆}$ yields Equation 31:

$$\text{tr }\;\left(\Sigma_{n^{⋆}}^{\text{bdry}}\right)\leq {\bar{F}}^{2n^{⋆}}\text{tr }\;\left(\Sigma_{0}^{\text{bdry}}\right)+\left(\Delta t^{2}\bar{r}+\bar{q}\right)\overset{n^{⋆}-1}{\underset{s=0}{\sum }}{\bar{F}}^{2s}.$$ (31)

Since $n^{⋆}\leq K_{\max}$ , $\alpha =\max\left\{1,{\bar{F}}^{2}\right\}$ , and $\Sigma_{0}^{\text{bdry}}⪯{\bar{\Sigma }}^{\text{upd}}$ , the stated uniform trace bound follows. The same upper bound applies to each boundary-vertex covariance trace. Summing over the $N_{B}$ vertices gives

$$J_{i}\left(t\right)\leq N_{B}\;\bar{p}\left(K_{\max}\right),$$

which proves uniform boundedness.

Proposition 2 Sufficient service-capacity condition for bounded uncertainty.

Suppose each maintained patch must be serviced at least once every $T_{\max}$ seconds to satisfy the boundedness condition in Proposition 1. The following result analyzes a conservative cyclic servicing abstraction of the receding-horizon scheduler, rather than the exact adaptive scheduler trajectory. Consider a single vehicle servicing a fixed active patch subset S⊆I(t) over one mission cycle, with nominal service duration $T_{\text{svc}}$ per patch. Let L(S) denote a conservative upper bound on the length of a feasible closed tour through the current service points ${\left\{r_{i}\left(t\right)\right\}}_{i\in S}$ , and let $v_{\text{eff}}>0$ be a conservative strictly positive lower bound on the vehicle’s net ground speed along the tour under the estimated flow field. If (Equation 32)

$$\frac{L\left(S\right)}{v_{\text{eff}}}+|S|T_{\text{svc}}\leq T_{\max},$$ (32)

then every patch in S is revisited within $T_{\max}$ , hence satisfying the boundedness condition of Proposition 1. This condition is sufficient but not necessary; the adaptive scheduler may still maintain bounded uncertainty in cases where this conservative closed-tour bound is violated.

Equivalently, for fixed L(S) , $v_{\text{eff}}$ , and $T_{\text{svc}}>0$ , a sufficient capacity bound is presented in Equation 33:

$$|S|\leq \left\lfloor \frac{T_{\max}-L\left(S\right)/v_{\text{eff}}}{T_{\text{svc}}}\right\rfloor .$$ (33)

Proof. The time required for the vehicle to complete one full service cycle over the patch set S is bounded by

$$T_{\text{cycle}}=\frac{L\left(S\right)}{v_{\text{eff}}}+|S|T_{\text{svc}}.$$

Under the cyclic servicing abstraction, each patch in S is visited once per cycle. Therefore, the maximum revisit time for any patch in S is at most $T_{\text{cycle}}$ . If Equation 32 holds, then $T_{\text{cycle}}\leq T_{\max}$ , so every patch in S is revisited within the maximum allowable interval required by Proposition 1. Rearranging Equation 32 gives the sufficient capacity bound in Equation 33.

Operationally, Propositions 1, 2 show how estimation accuracy, revisit timing, service duration, and vehicle mobility are coupled. Proposition 1 shows that patch uncertainty remains bounded when boundary measurements repeatedly reduce the uncertainty accumulated during unobserved advection within a maximum revisit interval. Proposition 2 translates this estimation requirement into a mission-level feasibility condition: the vehicle must be able to complete the tour and spend the required service time at each patch before the revisit deadline $T_{\max}$ is exceeded. Therefore, a larger active patch set, longer inter-patch travel distance, lower effective vehicle speed, or longer service time can violate the boundedness condition even if the estimator and controller are locally well designed.

### 3.4 Flow-aware gain-scheduled LQR tracking

After the scheduler selects the target patch $i^{⋆}$ , the controller tracks the corresponding patch-level reference

$$r\left(t\right)=r_{i^{⋆}}\left(t\right).$$

The reference $r_{i^{⋆}}\left(t\right)$ is generated from the current regularized boundary of patch $i^{⋆}$ , as defined in Section 3.2. When the centroid is admissible, this reference is the centroid of the selected patch; otherwise, it is the admissible fallback service point computed from the regularized boundary.

The controller is designed using a reduced-order planar kinematic model with additive estimated flow, as shown in Equation 34:

$$\dot{p}=u\left[\begin{array}{c} \cos\psi \\ \sin\psi \end{array}\right]+{\hat{v}}_{f}^{\;n}\left(p,t\right),\;\dot{u}=a,\;\dot{\psi }=\omega ,$$ (34)

where $p={\left[x,y\right]}^{⊤}$ is the vehicle position, u is the surge speed, ψ is the heading, a is the commanded surge acceleration, ω is the commanded yaw rate, and ${\hat{v}}_{f}^{\;n}\left(p,t\right)$ is the estimated inertial-frame flow interpolated from the flow map. The reduced-order tracking model is used only for guidance synthesis, while the estimator and simulation retain the planar marine-craft dynamics described by Fossen (2011).

Reference frame and tracking errors: A moving tangent–normal frame is attached to the selected reference. Let $\psi_{d}\left(t\right)$ denote the desired heading. When the reference velocity is available and nonzero, the desired heading is chosen as

$$\psi_{d}\left(t\right)=\text{atan}2\;\left({\dot{r}}_{y}\left(t\right),{\dot{r}}_{x}\left(t\right)\right).$$

When the reference is nearly stationary, $\psi_{d}\left(t\right)$ is chosen from the line-of-sight direction to the reference or from a filtered previous desired heading to avoid singularity. The tangent and normal vectors are as follows:

$$T\left(t\right)=\left[\begin{array}{c} \cos\psi_{d}\left(t\right)\\ \sin\psi_{d}\left(t\right) \end{array}\right],\;N\left(t\right)=\left[\begin{array}{c} -\sin\psi_{d}\left(t\right)\\ \cos\psi_{d}\left(t\right) \end{array}\right].$$

These vectors define a local moving tangent–normal tracking frame attached to the reference motion.

Let

$$e_{p}\left(t\right)=p\left(t\right)-r\left(t\right)$$

be the inertial position error. The path-relative tracking errors are presented in Equations 35–38:

$$e_{n}=N^{⊤}e_{p},$$ (35)

$$e_{t}=T^{⊤}e_{p},$$ (36)

$$e_{\psi }=\text{wrap }\left(\psi -\psi_{d}\right),$$ (37)

$$e_{u}=u-v_{d},$$ (38)

where $e_{n}$ is the cross-track error, $e_{t}$ is the along-track error, $e_{\psi }$ is the heading error, $e_{u}$ is the surge-speed error, and $v_{d}$ is the desired surge speed.

Energy-aware feed-forward speed: Energy awareness is incorporated through the feed-forward desired surge speed, rather than by modifying the quadratic LQR cost. When the estimated flow assists motion along the tangent direction, the desired surge speed is reduced according to Equation 39:

$$v_{d}=\max\;\left(v_{\min},\;v_{0}-k_{\text{tail}}\max\;\left(0,T^{⊤}{\hat{v}}_{f}^{\;n}\left(r,t\right)\right)\right),$$ (39)

where $v_{0}$ is the nominal desired surge speed in still water, $v_{\min}$ is the minimum desired surge speed, and $k_{\text{tail}}>0$ determines the strength of tail-flow compensation. This feed-forward shaping reduces the commanded water-relative motion when the estimated flow already contributes to motion in the desired tangent direction. It is an effort-reduction heuristic, not a full propulsion-power model.

The nominal feed-forward terms are denoted by

$$a_{d}={\dot{v}}_{d},\;\omega_{d}={\dot{\psi }}_{d}.$$

In the present implementation, $a_{d}=0$ unless a time-varying desired surge profile is imposed. For point-tracking or slowly moving patch references, $\omega_{d}$ may be set to zero or computed from a filtered desired-heading signal.

Linearized tracking-error model: Define the controller error state and input deviation as follows:

$$e={\left[\begin{array}{cccc} e_{n} & e_{t} & e_{\psi } & e_{u} \end{array}\right]}^{⊤},\;\delta u=\left[\begin{array}{c} a-a_{d}\\ \omega -\omega_{d} \end{array}\right].$$

Using the reduced-order kinematic model in Equation 34, the path-relative tracking errors satisfy nonlinear moving-frame error dynamics. Linearizing these dynamics about small errors,

$$e_{n}\approx 0,\;e_{t}\approx 0,\;e_{\psi }\approx 0,$$

and about the nominal operating condition $u\approx v_{d}$ , gives the local linear time-varying approximation (Equation 40):

$$\dot{e}=A\left(t\right)e+B\delta u+d_{f}\left(t\right).$$ (40)

The matrices are presented in Equation 41:

$$A\left(t\right)=\left[\begin{array}{cccc} 0 & -\kappa_{r}\left(t\right)v_{d} & v_{d} & 0\\ 0 & -k_{s} & 0 & 1\\ 0 & 0 & 0 & 0\\ 0 & 0 & 0 & 0 \end{array}\right],\;B=\left[\begin{array}{cc} 0 & 0\\ 0 & 0\\ 0 & 1\\ 1 & 0 \end{array}\right].$$ (41)

Here, $\kappa_{r}\left(t\right)$ is the local curvature of the reference direction. The scalar $k_{s}>0$ is an along-track relaxation gain introduced to reduce slow drift in the tangential error during point-tracking operation. The matrices above are obtained from a first-order linearization of the moving tangent–normal tracking-error dynamics about the nominal operating condition (Fossen, 2011; Breivik and Fossen, 2004). In particular, the terms involving $\kappa_{r}\left(t\right)v_{d}$ and $v_{d}e_{\psi }$ arise from the local curvature of the reference direction and the heading-error-induced lateral motion, respectively. For point-tracking or centroid-hold operation, the reference is treated locally as a moving point and $\kappa_{r}\left(t\right)=0$ .

The additive residual term is presented in Equation 42:

$$d_{f}\left(t\right)=\left[\begin{array}{c} N^{⊤}\left({\hat{v}}_{f}^{\;n}\left(r,t\right)-\dot{r}\right)\\ T^{⊤}\left({\hat{v}}_{f}^{\;n}\left(r,t\right)-\dot{r}\right)\\ 0\\ 0 \end{array}\right].$$ (42)

The term $d_{f}\left(t\right)$ represents flow- and reference-motion-induced drift. It is not used to compute the LQR gain directly; instead, it is treated as a bounded disturbance, while the gain is computed from $\left(A_{d}\left(k\right),B_{d}\right)$ . The feedback controller is designed from the local linear model, while the flow estimate also enters the feed-forward speed shaping in Equation 39.

Gain-scheduled LQR feedback law: The gain-scheduled LQR structure is used because the tracking problem involves coupled cross-track, along-track, heading, and surge-speed errors. Compared with independent proportional–integral–derivative (PID) loops, the LQR formulation provides a systematic way to penalize these coupled errors and the corresponding control effort through the weighting matrices $Q_{\text{LQR}}$ and $R_{\text{LQR}}$ . This is useful here because the local tracking model changes with the selected patch reference, desired surge speed, and estimated flow conditions. The resulting controller is computed on a reduced-order four-state error model. The local linear model is discretized at the fast-loop period Δt (Equation 43):

$$e_{k+1}=A_{d}\left(k\right)e_{k}+B_{d}\delta u_{k}+\Delta t\;d_{f}\left(k\right),$$ (43)

with Equation 44:

$$A_{d}\left(k\right)=I+\Delta t\;A\left(k\right),\;B_{d}=\Delta t\;B.$$ (44)

At each fast-loop sample k , the pair $\left(A_{d}\left(k\right),B_{d}\right)$ is frozen at the current operating point, and a discrete-time LQR gain is computed for the homogeneous part of Equation 43. The local quadratic objective is presented in Equation 45:

$$J_{\text{LQR},k}=\overset{\infty }{\underset{s=0}{\sum }}\left(e_{k+s}^{⊤}Q_{\text{LQR}}e_{k+s}+\delta u_{k+s}^{⊤}R_{\text{LQR}}\delta u_{k+s}\right),$$ (45)

where s is the prediction-step index measured from the current fast-loop sample k , $Q_{\text{LQR}}⪰0$ weights tracking error, and $R_{\text{LQR}}≻0$ weights control effort.

The frozen-time gain is obtained from the standard discrete-time LQR problem and associated discrete algebraic Riccati equation (Anderson and Moore, 2007). For the frozen pair $\left(A_{d}\left(k\right),B_{d}\right)$ , the feedback gain is presented in Equation 46:

$$K\left(k\right)={\left(R_{\text{LQR}}+B_{d}^{⊤}\Pi_{k}B_{d}\right)}^{-1}B_{d}^{⊤}\Pi_{k}A_{d}\left(k\right),$$ (46)

where $\Pi_{k}⪰0$ solves the discrete algebraic Riccati (Equation 47):

$$\begin{array}{cc} \Pi_{k} & =A_{d}{\left(k\right)}^{⊤}\Pi_{k}A_{d}\left(k\right)\\ & \;-A_{d}{\left(k\right)}^{⊤}\Pi_{k}B_{d}{\left(R_{\text{LQR}}+B_{d}^{⊤}\Pi_{k}B_{d}\right)}^{-1}B_{d}^{⊤}\Pi_{k}A_{d}\left(k\right)\\ & \;+Q_{\text{LQR}}. \end{array}$$ (47)

This produces a frozen-time, gain-scheduled LQR controller rather than a finite-horizon Riccati-based LQR.

The feedback law is as follows:

$$\delta u_{k}=-K\left(k\right)e_{k}.$$

Therefore, the commanded input is presented in Equation 48:

$$u_{\text{cmd},k}=\left[\begin{array}{c} a_{\text{cmd},k}\\ \omega_{\text{cmd},k} \end{array}\right]=\left[\begin{array}{c} a_{d}\\ \omega_{d} \end{array}\right]-K\left(k\right)e_{k}.$$ (48)

The commanded inputs are saturated according to actuator limits and applied to the full vehicle dynamics through the low-level actuation system. Recomputing K(k) as $v_{d}$ , $\kappa_{r}\left(t\right)$ , and the local reference geometry change allows the controller to remain matched to the current operating point, while the feed-forward speed shaping exploits favorable flow to reduce water-relative effort.

## 4 Results and discussion

This section evaluates the proposed uncertainty-aware multi-patch tracking framework using a data-replay experiment based on HF-radar surface-current estimates over a selected region of interest (ROI). The framework combines three coupled modules: uncertainty-aware patch-boundary propagation, service-event boundary correction with local flow-map covariance refinement, and a mission-level scheduler that selects the next patch to service using predicted uncertainty and travel effort.

### 4.1 Replay setup and evaluation metrics

**FIGURE 4:**
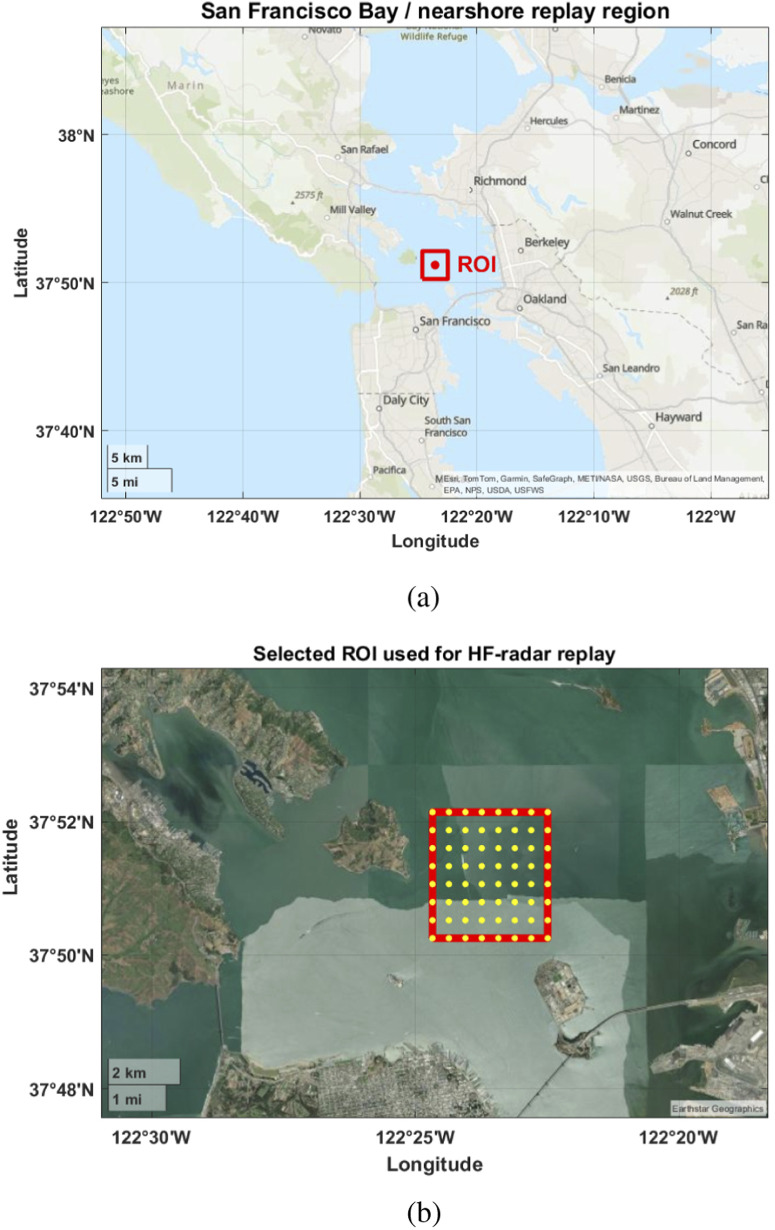
Study area for the HF-radar data replay. Basemap background from ARCGIS (2026). **(a)** San Francisco Bay/nearshore region covered using the UCSD HF-radar W500 product, with the selected replay region of interest (ROI) outlined in red. **(b)** Zoomed view of the ROI used in the experiments.

The data replay experiment uses gridded HF-radar surface-current estimates from the UCSD HF-radar W500 product for the San Francisco Bay/nearshore region. This product provides hourly snapshots of eastward and northward surface-velocity components on a fixed latitude–longitude grid (DOC/NOAA/NESDIS/OSPO, 2026). The study area and selected replay ROI are shown in Figure 4. A 7-day interval in February 2026 was used, yielding 169 hourly frames with an inter-frame spacing of 1 h . Because of environmental conditions, radar coverage, and quality-control filtering, some grid entries are missing at individual hourly frames.

To obtain a spatial region suitable for continuous advection and interpolation, we first searched for a compact ROI with consistently available HF-radar data over the full 7-day replay period. For each radar grid cell, temporal availability was computed as the fraction of hourly samples for which valid surface-current data were available. This produced an availability map over the radar domain. The ROI was then chosen as the largest contiguous square block of cells for which every cell exceeded an 80% availability threshold. This selection ensures that the replay domain is spatially compact and sufficiently well populated in time, so that patch-boundary advection and flow interpolation can be performed without frequent gaps or excessive extrapolation.

For the experiments reported here, this procedure yielded an 8×8 grid-cell ROI. The approximate spatial resolution is Δx≈500 m in the north–south (meridional) direction and Δy≈460 m in the east–west (zonal) direction, giving a total coverage of approximately 4.0 km×3.7 km .

Although the selected ROI has high temporal availability, a small fraction of entries remains missing. These gaps are filled using a Data Interpolating Empirical Orthogonal Functions (DINEOF)-style iterative low-rank reconstruction applied separately to the ROI time–space matrices for the eastward and northward velocity components (Alvera-Azcárate et al., 2011). This produces a fully defined replay flow field and is consistent with standard ocean-data gap-filling practice (Alvera-Azcárate et al., 2005).

During replay, the flow field is evaluated continuously by linear interpolation between the two nearest hourly HF-radar snapshots. Therefore, the replay contains temporal variation at the resolution supported by the hourly HF-radar product, while the vehicle and patch propagation are integrated at the smaller simulation time step Δt . For the hourly sampling interval of the HF-radar data, the updated replay experiments use a multi-hour horizon of $T_{\text{sim}}=10800$ , corresponding to 3 h , and therefore span multiple consecutive HF-radar frames.

### 4.2 Multi-patch tracking demonstration

**FIGURE 5:**
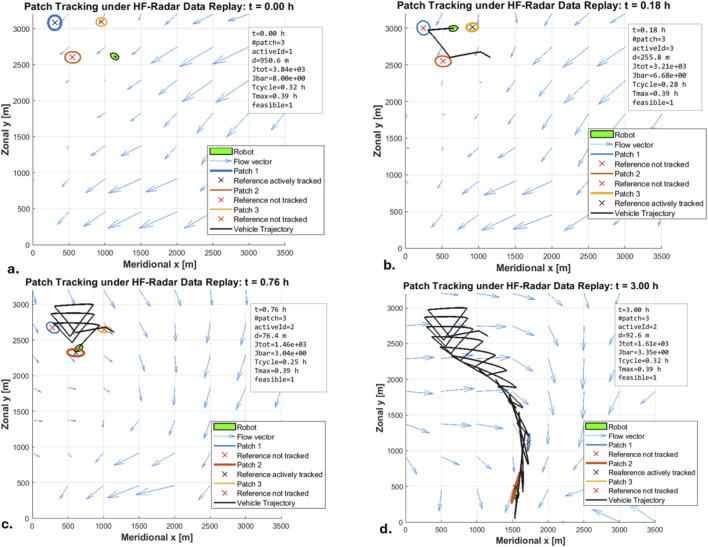
Multi-patch tracking replay under the HF-radar flow field. Snapshots are shown at **(a)** t=0.00 h , **(b)** t=0.18 h , **(c)** t=0.76 h , and **(d)** t=3.00 h .

**FIGURE 6:**
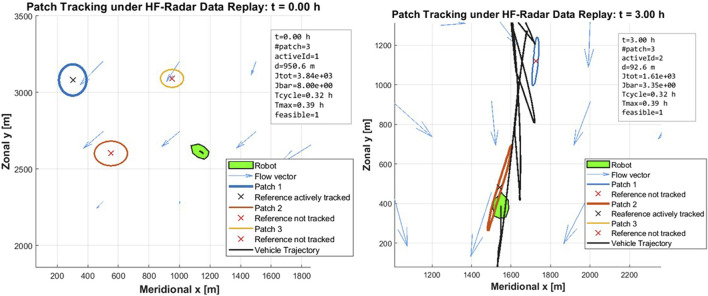
Zoomed Map of Figure 5a on the left and Figure 5d on the right.

We first evaluate the closed-loop framework in a three-patch replay using the spatially and temporally varying HF-radar flow field described in Section 4.1. Figure 5 shows snapshots of the vehicle, patch boundaries, local flow vectors, and active tracking target over the replay horizon. Figure 6 provides a zoomed comparison of the patch configuration at the beginning and end of the replay.

The vehicle is shown by the green marker, while the black curve denotes the vehicle trajectory. For clarity, the three patches are indicated in three different colors, and the vehicle trajectory is shown as a black line. For each patch, the marker indicates the patch-level reference point $r_{i}\left(t\right)$ , which is computed from the current regularized boundary as described in Section 3.2. The active patch reference selected by the scheduler is highlighted in black, while inactive patch references are shown in red. In addition, the mission parameters as the robot continues its mission are shown at the top-right corner of the four figures.

In the updated multi-hour replay, the snapshots are reported in hours to make the temporal scale of the HF-radar product explicit. The simulation begins at t=0 h , with the vehicle and three candidate patches initialized inside the selected ROI. As the replay progresses, the scheduler switches the active target according to the predicted uncertainty, revisit age, and travel-effort score. For example, the vehicle tracks different patch references at intermediate times and continues servicing the active set through t=3 h . This behavior illustrates the closed-loop interaction between patch propagation, scheduler target selection, and reference tracking over a replay horizon spanning multiple hourly HF-radar intervals.

**FIGURE 7:**
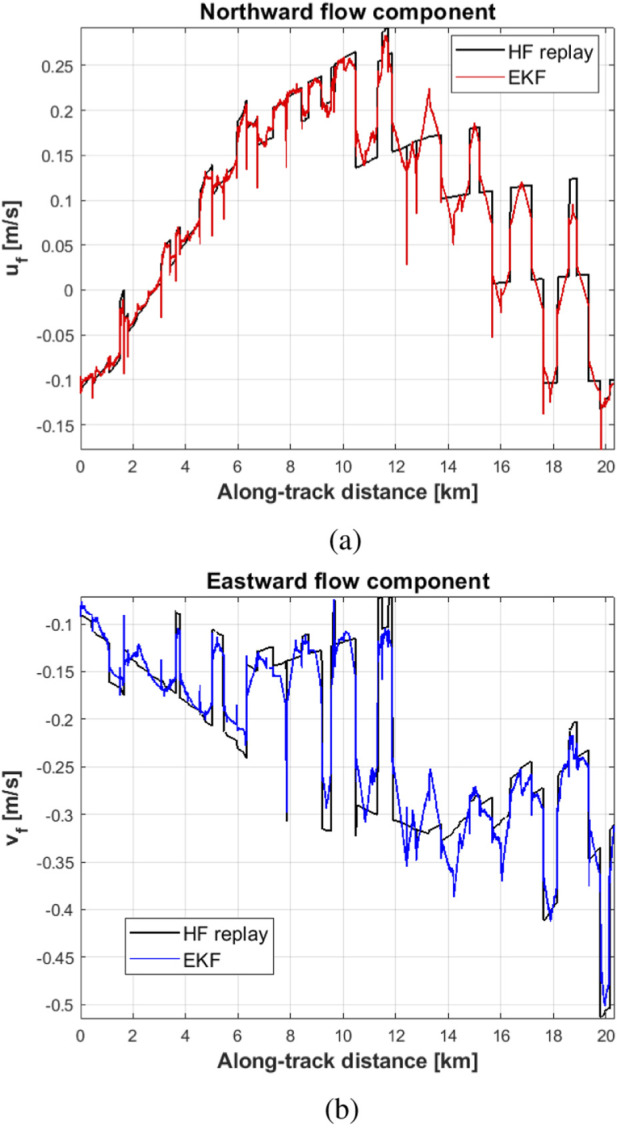
EKF flow-estimation performance along the vehicle trajectory. The northward and eastward flow components are plotted against along-track distance. Black curves denote the HF-radar replay values sampled along the traversed cells, and colored curves denote the EKF estimates. **(a)** Northward flow component $u_{f}$ . **(b)** Eastward flow component $v_{f}$.

During the same replay, the vehicle also estimates the local flow in the map cells that it traverses. Figure 7 compares the EKF-estimated flow components with the replay reference values along the vehicle trajectory. The flow-estimation error was evaluated only over traversed cells since those are the cells directly updated by the active-cell EKF. The EKF achieved component-wise root-mean-square errors of 0.015017 m/s in the northward component $u_{f}$ and 0.023067 m/s in the eastward component $v_{f}$ . The corresponding two-component vector RMSE was 0.0275 m/s . These results indicate that, in the replay setting, the filter was able to recover the local cell flow with small error along the vehicle trajectory.

### 4.3 Empirical boundedness under feasible revisit conditions

**FIGURE 8:**
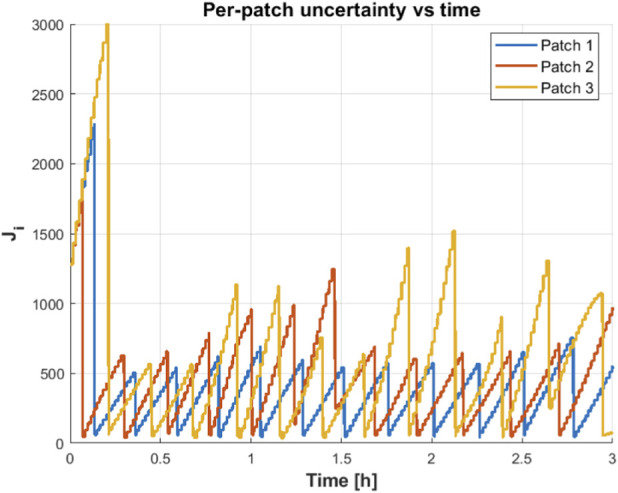
Per-patch uncertainty.

**FIGURE 9:**
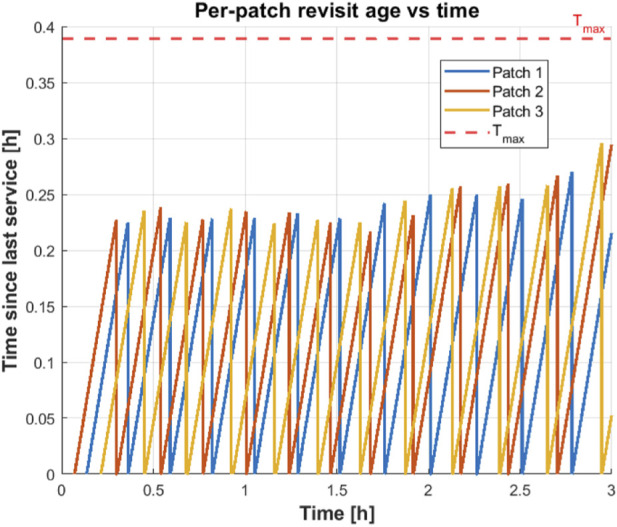
Per-Patch revisit duration.

**FIGURE 10:**
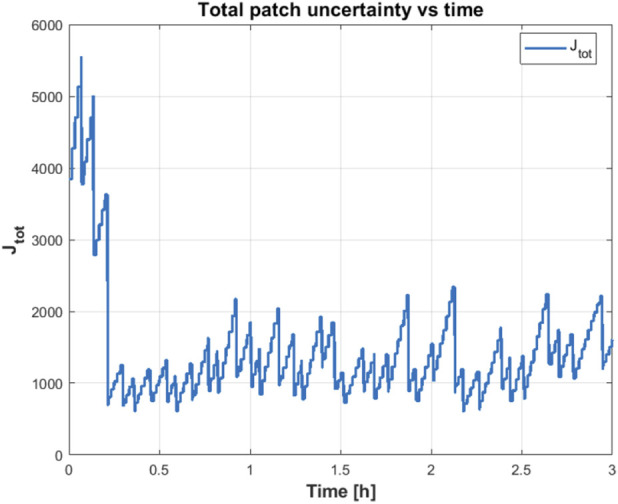
Overall total patch uncertainty.

To evaluate the boundedness behavior of the proposed framework, we considered the three-patch replay case designed to satisfy the revisit-time requirement associated with Proposition 1. The replay was run over a multi-hour horizon, and the time axes in Figures 8–10 are reported in hours.

Figure 9 shows the per-patch revisit ages. For all three patches, the revisit age remained below the imposed threshold $T_{\max}$ throughout the replay. Correspondingly, the per-patch uncertainty trajectories in Figure 8 exhibit the expected sawtooth behavior: uncertainty increases during unobserved advection and decreases after a service event when boundary measurements are fused.

The aggregate uncertainty,

$$J_{\text{tot}}\left(t\right)=\underset{i\in I\left(t\right)}{\sum }J_{i}\left(t\right),$$

is shown in Figure 10. After the initial transient associated with the first service cycle, $J_{\text{tot}}\left(t\right)$ remains bounded over the replay horizon rather than drifting upward without limit. This behavior is empirically consistent with Proposition 1, which states that bounded revisit time, bounded flow uncertainty, and finite post-service boundary covariance imply bounded patch uncertainty. The result is also consistent with Proposition 2, which relates this revisit requirement to the mission-level quantities: cycle time, vehicle speed, tour length, and service duration.

### 4.4 Effect of boundary service and local flow-map covariance refinement

We next examined how service events affect both patch-boundary uncertainty and local flow-map covariance. When the vehicle services a patch, local boundary measurements are fused with the propagated boundary estimate. This reduces the instantaneous boundary uncertainty of the serviced patch. In addition, when successive associated boundary detections are available, their displacement can be used as a local pseudo-measurement of boundary advection velocity, as described in Section 3.3.

**FIGURE 11:**
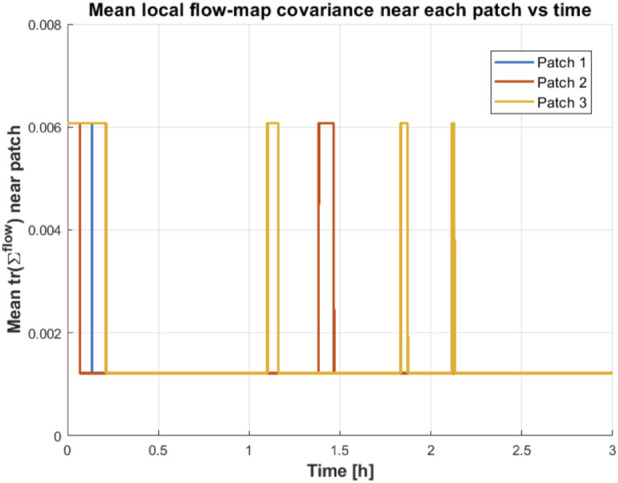
Mean trace of the local flow-map covariance in cells near serviced patch boundaries. The decrease after service events shows the effect of local map-covariance refinement.

Figure 11 shows the mean trace of the flow-map covariance in cells near the serviced patch boundaries. In the case with local map-covariance refinement, the mean covariance near serviced patches decreases after service events. This behavior is consistent with the intended role of the optional refinement step in Algorithm 3: boundary service does not only correct the currently observed patch boundary but can also reduce the flow-map covariance in nearby cells.

This reduction is important because the boundary covariance propagation in Equation 8 includes the following term:

$$\Delta t^{2}\Sigma^{\text{flow}}\left(b_{ij},t\right),$$

which represents uncertainty injected from the local flow map during advection. Therefore, reducing $\Sigma^{\text{flow}}\left(\cdot ,t\right)$ near serviced boundary regions lowers the subsequent uncertainty growth rate when patch vertices pass through those cells. In this way, a service event can reduce both the immediate boundary uncertainty and the future uncertainty accumulation during patch propagation.

### 4.5 Scheduler comparison in clustered and separated three-patch cases

After establishing boundedness behavior in the feasible three-patch replay, we evaluated the effect of the mission-level scheduler. Four scheduling policies were compared:

1. *Nearest patch*: selects the patch with the shortest instantaneous travel distance.
2. *Highest* J : selects the patch with the largest current boundary uncertainty.
3. *Round robin*: services the patches cyclically.
4. *Proposed predictive scheduler*: selects the patch using the predicted urgency-weighted uncertainty-per-effort score $\Phi_{i}$ .

The proposed scheduler uses predicted arrival uncertainty, travel-effort estimates, reachability checks, hysteresis, and minimum-service logic.

We first considered a clustered three-patch configuration, where the distances between patches are small, as shown in Figure 5. In this case, the highest- J strategy achieved the lowest uncertainty. This behavior is expected. When the patches are closely spaced, travel-effort differences between candidate patches become small, so the advantage of a cost-aware predictive policy is reduced. In that regime, a scheduler that always services the currently most uncertain patch can be highly effective. The clustered case, therefore, serves as a useful stress test, showing that the proposed scheduler is not automatically dominant in every geometry, especially when travel cost is not a strong differentiating factor.

To better evaluate the benefit of cost-aware scheduling, we then increased the spatial separation between the patches. In this more demanding geometry, the proposed scheduler better captured the intended tradeoff between uncertainty reduction and travel effort. The separated-case comparison is summarized in Table 2.

**TABLE 2:** Scheduler comparison for the separated three-patch replay case.

Scheduler	Mean patch uncertainty	Energy proxy	Fraction of patch-time below $J_{th}$
Proposed	**811.95**	**17.89**	0.2668
Highest J	860.68	18.85	**0.2715**
Nearest	5297.71	135.60	0.1040
Round robin	5297.76	133.50	0.1040

Bold values indicate the best-performing scheduler for the corresponding metric.

The results show two important trends. First, the proposed scheduler achieved the lowest mean patch uncertainty and the lowest realized energy proxy. These are the two metrics most directly aligned with the cost-aware mission objective, and they indicate that the predictive urgency–effort score can translate into a measurable mission-level advantage when inter-patch travel is non-negligible. Second, the nearest-patch and round-robin policies performed substantially worse, producing much larger uncertainty and higher control effort. This indicates that simple distance-only or cyclic scheduling is not sufficient when patch uncertainty grows during unobserved advection.

The comparison with the highest- J baseline is particularly informative. In the separated three-patch case, highest J achieved a slightly larger fraction of patch-time below $J_{th}$ than the proposed scheduler. This metric is defined as the fraction of all patch-time samples for which $J_{i}\left(t\right)\leq J_{th},$ so a larger value indicates that patch uncertainty remains below the prescribed threshold for a greater portion of the mission. The advantage of highest J on this metric is modest, approximately 0.0047, and is consistent with the fact that this baseline directly prioritizes the patch with the largest instantaneous uncertainty. In contrast, the proposed scheduler accepts a small loss in this threshold-based metric while reducing both mean uncertainty and travel/control effort.

The replay results support three observations. First, when the revisit requirement is satisfied, the propagation–service loop exhibits the bounded sawtooth uncertainty behavior predicted by Proposition 1. Second, service-driven local flow-map covariance refinement reduces uncertainty in visited regions, thereby reducing subsequent uncertainty injection during patch propagation. Third, the benefit of the proposed predictive scheduler becomes more apparent as the distance between patches increases. In the separated case, it achieved lower mean uncertainty and lower energy proxy than the strongest myopic baseline while also outperforming nearest-patch and round-robin scheduling by a large margin.

## 5 Conclusion and future work

This study presented a framework for uncertainty-aware persistent tracking of advected surface patches under time-varying currents. The method comprises covariance-aware patch propagation, intermittent boundary fusion during service, and mission-level target selection over multiple patches. The theoretical contribution established that if each patch is serviced within a bounded revisit interval, then the corresponding patch uncertainty remains uniformly bounded. In addition, a complementary feasibility condition related to the revisit deadline, effective vehicle speed, the travel distance over the patch set, and the service duration was explained. Together, these results provide a principled link between estimator uncertainty, service timing, and mission design. The analysis also provides a conservative sufficient capacity condition relating the number of maintained patches to tour length, effective speed, revisit deadline, and service duration.

To further evaluate our system, a comparison analysis was carried out with three other known strategies, namely, nearest-patch, round robin, and highest- J . The results show that the highest- J baseline can outperform the proposed scheduler when the patches are closely clustered. This is expected because travel-cost differences between candidate patches are small in that regime. However, when patches are spatially separated, which is closely related to the typical ocean environment, the proposed scheduler achieved lower mean patch uncertainty and lower realized control-effort proxy than the strongest myopic baseline. It also outperformed the nearest-patch and round-robin scheduling strategies. This indicates that the predictive cost-aware formulation becomes advantageous when inter-patch travel is non-negligible and persistent service must be delivered over a distributed set of targets.

Ongoing works include the following: first, the scheduler can be improved by ranking patches using expected uncertainty reduction per travel cost, rather than arrival uncertainty alone, so that the selection policy more directly reflects the value of service. Second, the study can be further strengthened by incorporating additional truth-referenced experiments, including simulated ground-truth boundary error measures and, ultimately, field trials with real vehicle sensing. Finally, extending this framework to accommodate multiple vehicles, larger patch sets, and heterogeneous service requirements would further increase its practical relevance for persistent ocean monitoring. Overall, the results suggest that combining uncertainty-aware estimation with cost-aware decision-making is a viable path toward persistent autonomous monitoring of evolving marine surface phenomena.

## Data Availability

Publicly available datasets were analyzed in this study. These data can be found here: https://coastwatch.pfeg.noaa.gov/erddap/griddap/ucsdHfrW500.html.
